# Nurses' workload and its relation with physiological stress reactions^1^

**DOI:** 10.1590/0104-1169.3292.2503

**Published:** 2014

**Authors:** Rita de Cássia de Marchi Barcellos Dalri, Luiz Almeida da Silva, Aida Maria Oliveira Cruz Mendes, Maria Lúcia do Carmo Cruz Robazzi

**Affiliations:** 2 Post-doctoral fellow, Escola de Enfermagem de Ribeirão Preto, Universidade de São Paulo, WHO Collaborating Centre for Nursing Research Development, Ribeirão Preto, SP, Brazil; 3 PhD, Adjunct Professor, Universidade Federal de Goiás, Jataí, GO, Brazil; 4 PhD, Coordinator Professor, Escola Superior de Enfermagem de Coimbra, Coimbra, Portugal; 5 PhD, Full Professor, Escola de Enfermagem de Ribeirão Preto, Universidade de São Paulo, WHO Collaborating Centre for Nursing Research Development, Ribeirão Preto, SP, Brazil

**Keywords:** Nursing, Work, Occupational Health Nursing, Occupational Diseases, Nursing Service, Hospital

## Abstract

**OBJECTIVE:**

to analyze the relation between the workload and the physiological stress reactions among nurses working at a hospital service.

**METHODS:**

cross-sectional, correlational, quantitative study, involving 95 nurses, in 2011 and 2012. Spearman's bivariate Correlation Test was used.

**RESULTS:**

most subjects are female, between 23 and 61 years old and working between 21 and 78 hours per week. The most frequent physiological reactions were back pain, fatigue/exhaustion, stiff neck and stomach acidity, with 46.3% of the subjects presenting low and 42.1% moderate physiological stress responses. No correlation was found between the workload and the physiological stress responses.

**CONCLUSION:**

although most of the nurses work more than 36 hours/week, physiologically, they do not present high reaction levels in response to stress. These workers deal with conflicts in the vertical and horizontal relations between professionals, family members and patients. In that sense, taking care of professionals who offer health services can be a fundamental strategy, as good user care mainly depends on healthy teams.

## Introduction

The current labor conditions involve production and service models with accelerated and intensified work characteristics. The models determine increased productivity, through the combination of the work rhythm, responsibility burden and reduction of rest intervals in the work journey. These facts can lead to a progressive occupational risk trend, which can give rise to chronic effects in the workers' health. In that sense, the work journey represents an important dimension of the job quality, affecting the occupational safety and health, in the personal and family aspects as well as in the work organization inside the institution^(^ 1 ^)^.

The work journey can turn into an element that causes exhaustion and suffering for the workers; when the organizational context triggers suffering, individuals aim to develop defense mechanisms to try and reduce it. In case of increased conflicts and deadlocks between the workers and the organization, however, when they are no longer able to give an outlet to their desires and creative/inventive processes, they end up getting ill^(^ 2 ^)^ and the organizational environments can become stressful for the workers. On the other hand, work is a form of being for humans and they can get remuneration and satisfaction through their work, avoiding or mitigating stress situations^(^ 3 ^)^.

Stress can be acute or chronic, and the consequences of high chronic stress levels are perceived through absenteeism, productivity drop, demotivation, interpersonal difficulties, different physical illnesses, depression, anxiety and unhappiness in the personal sphere. In the work sphere, the consequences of stress can also include low spirits, lack of involvement with work and the organization, frequent absences and delays, excessive visits to the medical clinic and medication dependence^(^ 4 ^)^.

In nursing work, the concern with these professionals' suffering and pleasures emerges, arousing questions about how they are able to bear exhausting situations, mainly due to the constant contact with suffering, pain, death and so many other feelings and reactions the disease process triggers. Nursing has worked to attend to human beings and, therefore, gain knowledge and scientific principles that support their practice. Nevertheless, the nurses' work conditions lead to physical and emotional exhaustion^(^ 5 ^-^ 6 ^)^.

In parallel, it was observed that the stress experience is multifaceted, with a reasonable range of dimensions that can contribute to the nurses' work problems. This experience indicates the need for intervention problems that aim to "combat" the different work problems and include various strategies, as it is totally different to outline an intervention aimed at improving the relation with the patients and another that helps the workers to better manage the stress associated with the "fear of committing errors"^(^ 7 ^)^.

Stressed nurses are more susceptible to the occurrence of work-related accidents and diseases and can also develop their activities inefficiently, certainly resulting in negative consequences for the attended individuals and/or population^(^ 8 ^)^. In addition to this problem, there is the large hour load the health workers, including the nursing professionals, tend to accomplish, making them work excessively.

The work overload seems to favor mental and/or physical illnesses in health workers, besides facilitating the occurrence of absenteeism, occupational accidents, medication errors, exhaustion, work overload and absence of leisure^(^ 9 ^)^. To overcome the adversities of their work, the nurses seek motivation, such as money and knowledge, to keep up a double work journey, challenging the extrinsic and intrinsic factors that constantly emerge^(^ 5 ^)^.

Although the literature indicates the presence of stress and exhaustion among nurses due to different reasons, including overtime and involving physical and/or mental symptoms due to the work they perform^(^ 6 ^)^, with work overload and lack of time even to rest^(^ 5 ^)^, no studies have been identified that address the physiological reactions caused by stress, specifically related to the hour load of nurses working in hospital contexts.

In view of this knowledge gap about the theme, which can support improvements in the nursing work conditions and in the quality of patient care, this study aimed to analyze the relation between work burden and physiological stress reactions among hospital nurses.

## Method

Cross-sectional, quantitative correlation study. Undertaken at a public hospital located in Ribeirão Preto, São Paulo, Brazil between the second semester of 2011 and the first semester of 2012.

The study population consisted of 131 nurses, working in all sectors and work shifts. Due to the population size, the decision was made to work with the complete universe of workers, without any sample size calculation. The following selection criteria were established: being available on the dates set for the data collection. The nurses could be hired by the hospital's Support Foundation or by the State, underlining that these contracts are independent. In the first situation, the nurses were not admitted through a public exam and, in the second, they did take part in this exam. After the application of the selection criteria, the population consisted of 95 subjects, that is, 72.5% of the total population.

The data collection instrument for the personal and professional characteristics consisted of 12 questions, focused on the variables that were aimed at identifying the workers and their professional activity. The diagnosis of the physiological stress response reactions was assessed using the Inventory of Physiological Responses to Stress, previously validated for Brazil^(^ 10 ^)^, which consists of 39 stress-related symptoms, to be scored on a Likert scale [never (1), rarely (2), sometimes (3), frequently (4) and constantly (5)]; the sum indicates the physiological symptoms in response to stress: 40 - 75 low symptoms; 76 - 100 moderate symptoms, 101 - 150 high symptoms and >150 excessive stress response symptoms. The symptoms measured were tension headache, migraine (vascular headache), stomach ache, increased blood pressure, cold hands, stomach acidity, fast and superficial breathing, diarrhea, palpitations, trembling hands, burps, flatulence, greater urge to urinate, sweating hands and/or feet, oily skin, fatigue/feeling of exhaustion, breathlessness, dry mouth, trembling of the hands, back pain, stiff neck, chewing gum, teeth gnashing, constipation, feeling of tight chest/heart, dizziness, nausea/vomiting, menstrual pain, skin spots, extra-systoles, colitis, asthma, indigestion, high blood pressure, hyperventilation, arthritis, skin eruption, bruxism/jaw pain and allergy. To assess the reliability of the instrument, internal consistency analysis was applied using Cronbach's alpha, resulting in α= 0.900.

At the workplace, the primary author provided the nurses with orientations on the proposed study objective and the data collection procedures, in compliance with the guidelines of Resolution 196/96- CNS^(^ 11 ^)^, in force at the time of the approval and data collection. The study received approval from the Scientific Council of the *Centro de Estudos de Emergência em Saúde* at the study hospital and from the Research Ethics Committee of a higher education institution under protocol 1272/2010. The subjects read the Free and Informed Consent Form and the data collection started after two copies of the form had been signed. The primary author filed one of the copies and the research participant received the author.

First, the data were included in the software *MS-Excel* (2007) with dual input and later validated. Then, they were exported to the software* Statistical* *Package for Social Science *(SPSS^(c))^ version 19.0 for descriptive and inferential statistical analysis of the data. The normal distribution of the measures was verified using the *Kolmogorov-Smirnov* test and, as this was not confirmed, the bivariate form of *Spearman's *Correlation test was applied to check for correlations between the weekly workload and the physiological stress reactions. For this model, the significance level was set at α= 0.05.

## Results

Among the 95 subjects, 85 (89.4%) were female. The age ranged between 23 and 61 years and, regarding the marital status, 42 (44.2%) nurses were single and 41 (43.2%) were married or lived with a partner. Concerning the employment bonds, 76 (80%) subjects had only one, 17 (17.9%) two and two nurses three. As regards the type of job contract, 27 (28.4%) nurses were hired by the hospital's Support Foundation, 65 (68.4%) were state-hired public servant and only three were hired by the Foundation and the State.

The weekly workload ranged between 21 and 78 hours, with a median of 42 hours. It is emphasized that the subjects' workload was 36 hours/week for those hired by the Foundation and 30 hours/week for the state-hired public servants. As regards the return from holidays, on the date of the data collection, 71 (74.7%) nurses had returned more than 30 days earlier and 24 (25.3%) less than 30 days, which justifies some answers of a weekly workload of less than 30 hours.

**Figure 1 -:**
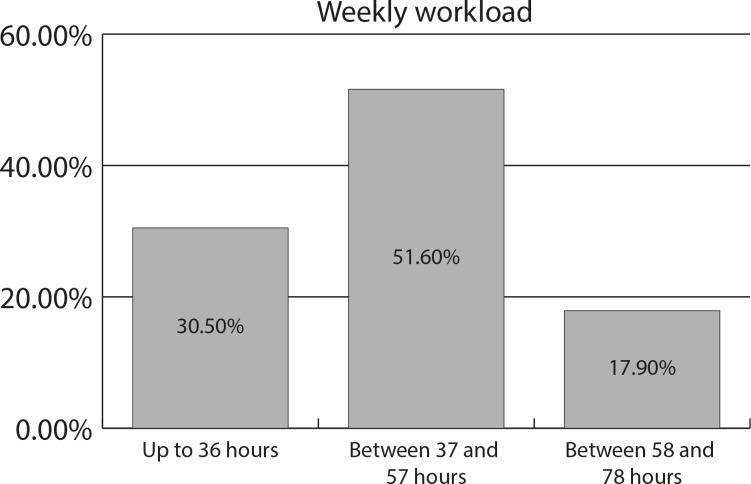
Percentage distribution of nurses at the public hospital according to weekly workload. Ribeirão Preto, SP, Brazil, 2012 (n=95)

Figure 1 displays the nurses' distribution according to the weekly workload.

**Figure 2 -:**
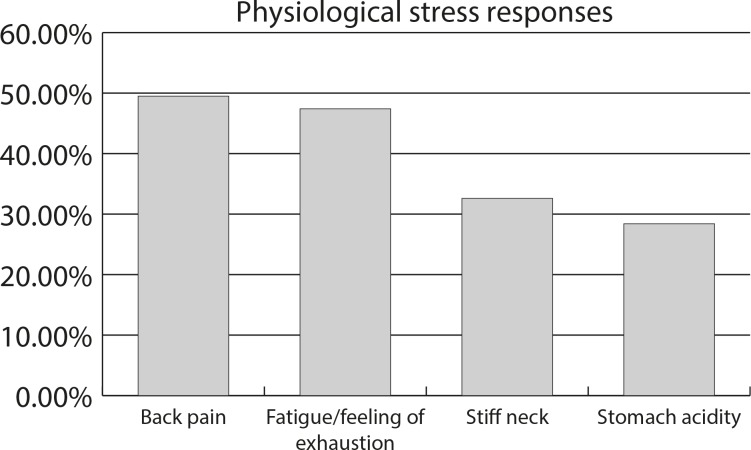
Physiological stress responses among the nurses at the public hospital. Ribeirão Preto, SP, Brazil, 2012 (n=95)

Figure 2 below shows the most significant physiological stress reactions, obtained through the Inventory of Physiological Stress Reactions^(^ 10 ^)^.

**Figure 3 -:**
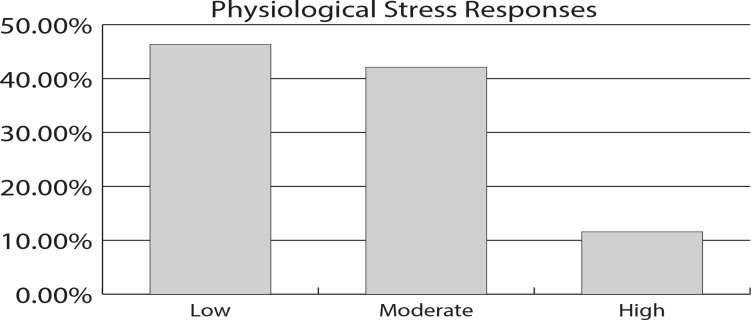
Classification of physiological stress responses among nurses at the public hospital according to the obtained scores. Ribeirão Preto, SP, Brasil, 2012 (n=95)

Figure 3 below shows the classification of the physiological stress responses according to the scores on the Inventory of Physiological Stress Reactions^(^ 10 ^)^.

**Figure 4 -:**
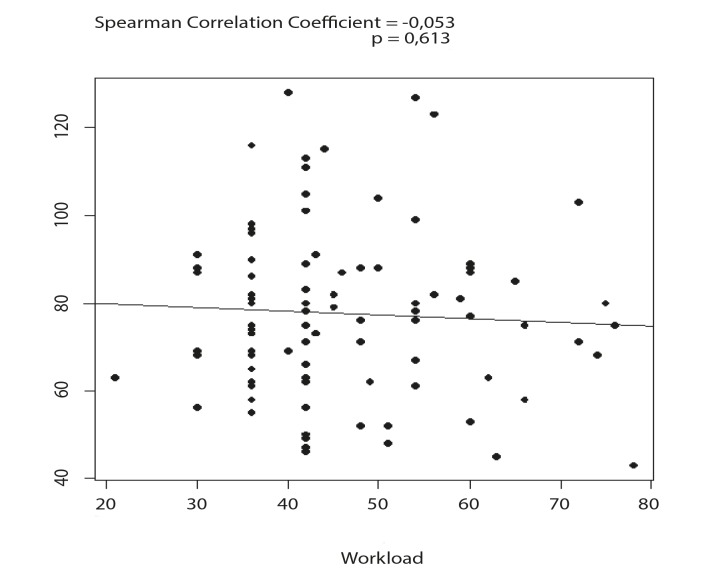
Dispersion diagram of weekly workload and physiological stress responses among the nurses at the public hospital. Ribeirão Preto, SP, Brazil, 2012 (n=95)

Spearman's Correlation test was used to check for correlations between the weekly workload and the physiological stress responses and did not show statistical evidence that prove the existence of this correlation, as shown in Figure 4.

## Discussion

Most of the subjects are female (89.4%), which shows that nursing remains an essentially female profession, as shown in the course of history^(^ 12 ^)^.

As regards the number of employment bonds, although 80% of the subjects had only one bond, the mean weekly workload was 46.2 hours, showing that the professionals work overtime at the same institution where they are professionally active. Only two nurses indicated three bonds and presented moderate physiological stress responses.

Federal Nursing Council (COFEn) Resolution 293/2004 determines that, to elaborate the monthly nursing staff scale, the workload should be 36 hours/week for care activities and 40 hours/week for administrative activities. In the Federal Chamber, Bill 2.295/2000 is under discussion, which establishes a maximum work journey of 30 hours per week for nurses, nursing technicians and auxiliary nurses^(^ 13 ^)^.

According to the International Labor Organization (ILO), about 22% of the global workforce, equivalent to approximately 614.2 million workers, work more than 48 hours per week. In Brazil, in 2008, 33.7% of the workers showed a work journey of more than 44 hours per week and 19.1% worked more than 48 hours per week^(^ 1 ^)^.

The nurses' professional stress reveals its importance, increasingly leading to exhaustion in the profession. One of the most frequent problems in a literature review about health changes due to excess work among health workers was occupational stress^(^ 9 ^)^.

The present study data, however, show that most of the nurses work their normal work hours and overtime at the same institution, which can favor a better adaptation to that sector, avoiding the stress that often results from a change of workplace during the daily work hours. When they remain at the same place, the nurses know the reality of the shift they assume, that is, they know the nursing technicians and auxiliary nurses subordinated to them, the medical team and other professionals on duty, the number and conditions of the patients under their responsibility, the availability of material and equipment, among others.

Results different from the above were presented in a research undertaken with nurses working at army hospitals in Taiwan, which detected an excessive workload as the main source of stress among them^(^ 14 ^)^.

Each person has a particular form of reacting to the stimuli of life and, therefore, also has different exhaustion thresholds due to stress. According to each person's viewpoint on reality, valuation of the past or future perspectives, the stress responses can vary^(^ 15 ^)^, a highly relevant factor in the emergence of diseases or not.

Nursing workers present an experience marked by occupational accidents, diseases, disability, absenteeism and abandonment of the profession^(^ 16 ^)^, factors that produce stress, but the subjects do not always know what to use as defense mechanisms.

Among the responses reported in this study, back pain was the most mentioned. This result coincides with the literature review involving nursing workers, which demonstrated that the prevailing diseases in these professionals were musculoskeletal^(^ 17 ^)^. Another study of nursing professionals working at the surgery center found musculoskeletal symptoms in the lumbar region (20.4%) in these workers^(^ 18 ^)^.

Also regarding the reactions, a study of Iranian nurses showed that they are exposed to high stress levels and the prevalence of musculoskeletal injuries was high, i.e. 89.9% of them presented some type of these injuries in the last 12 months before the research; lumbar complaints were the most commonly mentioned problems (6.8%)^(^ 19 ^)^.

Inappropriate environments, badly organized activities, little valuation of workers, unsatisfactory participation in decisions, excessive demand, low salaries and repetitive work, which favors incorrect postures in undesirable work situations, are elements that can favor illness among health professionals^(^ 9 ^)^. A study of nurses found that the stressors and measures to cope with these situations need to be investigated in their work environment, promoting benefits not only for the workers, but for all the individuals they attend to^(^ 20 ^)^.

The individuals try to find motives for satisfaction and accomplishment in their work. In the performance of their functions, but mainly when confronted with adverse situations, the organism tries to maintain its balance, using forms of adaptation.

In the health area, dealing directly with human lives, the professionals' performance comes with expectations of high competency and accountability levels in their work. Thus, studies and actions to improve the professionals' work conditions and quality of life are increasingly frequent^(^ 21 ^)^.

A study to investigate nursing workers' level of resilience, aiming to discover these professionals' strengths and weaknesses in view of the adversities they are submitted to, showed their excessive control of impulses and difficulties in the regulation of emotions, demanding great energy, as they do not externalize their emotions, especially in the work environment, justifying the high stress level found among the subjects^(^ 22 ^)^.

In that sense, taking care of professionals who offer health services can be a fundamental strategy, as good user care mainly depends on healthy work teams.

## Conclusion

As verified, most of the nurses studied work more than 36 hours/week but, physiologically, did not present high stress responses. Therefore, no statistical evidence was found that proves the existence of correlations between the weekly workload and the physiological stress responses among these subjects.

These findings stimulate the continuity of research in search of explanations, like the nurses' use of coping strategies for example, through the financial stimulus gained from overtime, favoring better living conditions for them and their relatives and satisfaction and pleasure in the activities they develop.

There is a need for further research to deepen the relation between workload and physiological stress responses among nurses, reminding that, although the excessive work hours did not give rise to high physiological stress responses among the subjects, can favor the occurrence of problems in patient care.

The study comes with limitations, as it involved a sample of 95 hospital nurses which, although representative, may not be similar to the multiple health contexts identified in Brazil.
